# A study on the antioxidant and antimicrobial activities in the chloroformic and methanolic extracts of 6 important medicinal plants collected from North of Iran

**DOI:** 10.1186/s13065-020-00683-5

**Published:** 2020-04-25

**Authors:** Zahra Hadadi, Ghorban Ali Nematzadeh, Somayeh Ghahari

**Affiliations:** 1 Department of Plant Breeding, Sari Agricultural Sciences and Natural Resources University, Sari, Iran; 2 Sari University of Agricultural Sciences and Natural Resources, Genetics and Agricultural Biotechnology Institute of Tabarestan (GABIT), Sari, Iran

**Keywords:** Antibacterial activities, Antifungal effects, Antioxidant activities, Plant extracts

## Abstract

**Background:**

As possible sources of natural bioactive molecules, the plant essential oils and extracts have been used globally in new antimicrobial compounds, food preservatives, and alternatives to treat infectious disease.

**Methods:**

In this research, the antimicrobial activities of chloroformic and methanolic extracts of *Sophora flavescens*, *Rhaponticum repens*, *Alhagi maurorum*, *Melia azedarach*, *Peganum harmala*, and *Juncus conglomeratus* were evaluated against 8 bacteria (*S. aureus*, *B. subtilis*, *R. toxicus*, *P. aeruginosa*, *E. coli*, *P. syringae*, *X. campestris*, *P. viridiflava*) and 3 fungi (*Pyricularia oryzae*, *Fusarium oxysporum* and *Botrytis cinerea*), through disc diffusion method. Furthermore, the essential oils of plants with the highest antibacterial activity were analyzed utilizing GC/MS. Moreover, the tested plants were exposed to screening for possible antioxidant effect utilizing DPPH test, guaiacol peroxidas, and catalase enzymes. Besides, the amount of total phenol and flavonoid of these plants was measured.

**Results:**

Among the tested plants, methanolic and chloroformic extracts of *P. harmala* fruits showed the highest antibacterial activity against the tested bacteria. Besides, the investigation of free radical scavenging effects of the tested plants indicated the highest DPPH, protein, guaiacol peroxidase, and catalase in *P. harmala*, *M. azedarach*, *J. conglomeratus* fruits, and *J. conglomeratus* fruits, respectively. In addition, the phytochemical analysis demonstrated the greatest amounts of total phenolic and flavonoid compositions in *J. conglomeratus* and *P. harmala*, respectively.

**Conclusion:**

The results indicated that these plants could act as a promising antimicrobial agent, due to their short killing time.

## Introduction

The plant essential oils and extracts, considered as possible sources of natural bioactive molecules, have been utilized globally in new antimicrobial compounds, food preservatives, and alternatives to treat infectious disease [1]. There are many researches about the antibacterial and antifungal activities of plant extracts and essential oils [2–6]. For example, Srinivasan et al. [7] measured the antimicrobial activity of 50 medicinal plants including *Eucalyptus globulus*. The results showed that *Eucalyptus globulus* had antimicrobial activity versus *Chromobacterium*, *Escherichia coli*, *Klebsiella pneumonia*, *Enterobacter faecalis*, *Pseudomonas aeruginosa*, *Proteus mirabilis*, *Salmonella partyphy*, *S. typhi*, *Bacillus subtilis*, and *Staphylococcus aureus* bacteria and did not show any antifungal activity on the tested fungus. Nagata et al. [8] investigated the antimicrobial activity of macrocarpals, phloroglucinol derivatives contained in Eucalyptus leaves, versus a diversity of bacteria containing oral bacteria. Among the tested bacteria, *P. gingivalis* presented the maximum sensitivity to macrocarpals. Furthermore, its trypsin-like proteinase activity and binding to saliva-coated hydroxyapatite beads were inhibited by macrocarpals. Hayet et al. [9] evaluated the antibacterial activities of ethyl acetate, chloroform, butanol and methanol extracts of *peganum harmala* leaves against some pathogens containing 11 g-positive and 6 g-negative bacteria, among which methanol and chloroform extracts exhibited a higher antibacterial activity versus gram-positive than gram-negative bacteria. Han and Guo [10] investigated the antibacterial activity of *Angelica sinensis* extract (AE), *Sophora flavescens* extract (SE), and herb pair *A. sinensis* and *S. flavescens* extract (HPE), according to the result of which HPE had strong antibacterial activity on *Escherichia coli*, *Staphylococcus aureus*, *Shigella castellani*, and *Chalmers*. Besides, SE was moderately active to *E. coli*. Moreover, Sen and Batra [11] examined the antimicrobial activity of ethanol, methanol, petroleum ether and water extracts of *Melia azedarach* L. leaves versus 8 human pathogens including *Staphylococcus aureus*, *Bacillus cereus*, *Pseudomonas aeruginosa*, *Escherichia coli*, *Aspergillus flavus*, *Aspergillus niger*, *Fusarium oxisporum*, and *Rhizopus stolonifera*. All the extracts indicated considerable activity versus all pathogens; however, the alcoholic extract exhibited the maximum inhibitory concentration versus all the microorganisms. Ahmad et al. [12] studied the antibacterial effect of *Alhagi maurorum* leaves extract and showed that the crude extract, chloroform, and ethyl acetate fractions had prominent effects, giving over 80% inhibition versus *Bacillus anthrax*. The crude extract displayed 80% inhibition versus *Shigella dysenteriae*. Similarly, the ethyl acetate and crude extract acted well versus *Salmonella typhe* by 78.35% and 76.50% inhibition respectively.

Furthermore, antioxidants helped to prevent cancer or heart diseases, as they could act as scavengers of free radicals and neutralized the damaging reactive free radicals in body cells before they could cause protein and lipid oxidation and decrease potential mutation [13]. Generally, plants include considerable extents of phytochemical antioxidants such as flavonoids, phenolics, carotenoids, and tannins, which can be utilized to scavenge the extra free radicals existing in the body [14]. Many researches have reported the antioxidant effect of essential oils and plant extracts. For example, Hayet et al. [9] examined the antioxidant activity of ethyl acetate, chloroform, butanol and methanol extracts of *Peganum harmala* leaves, demonstrating that methanol extract had the highest antioxidant activity. Nesrin and Tolan [15] proved the antioxidant effect of *Hyssopus officinalis*; however, it was lower than butylated hydroxytoluene and ascorbic acid. Ahmad et al. [12] indicated that extracts/fractions from *Alhagi maurorum* leaves displayed powerful radical scavenging activity, probably because of the existence of phenolic compounds in the plant.

The main aim of the present work was to study the chemical composition, antioxidant effects, and antimicrobial activities, while doing the phytochemical analysis of some important medicinal plants.

## Materials and methods

### Plant materials

The plants studied in this research are displayed in Table 1. All plants were collected from the research field of Sari Agricultural and Natural Resources University (SANRU), located at 53º 04′ E and 36º 39′ N (Iran), and identified from flora resources. A botanist authenticated the samples (different parts of the mentioned plants) and the voucher specimen deposited in the laboratory (Table 1).

**Table 1:** Characteristics, DPPH radical scavenging activity, Total phenol and flavonoid content of the investigated plants

Scientific name	Family	Parts of sample	Voucher specimen no.	IC_50_ (µg mL^−1^)	Total phenol content	Total flavonoid content
*S. flavescens*	Fabaceae	Aerial	966,510,282	6.12 ± 0.77	39.07 ± 0.01	69.39 ± 0.01
*R. repens*	Asteraceae	Aerial	966,510,574	6.94 ± 1.12	24.72 ± 0.03	68.86 ± 0.03
*A. maurorum*	Fabaceae	Aerial	966,510,515	7.87 ± 1.09	45.43 ± 0.02	146.71 ± 0.02
*M. azedarach*	Meliaceae	Fruit	966,510,063	11.02 ± 1.36	21.96 ± 0.00	48.68 ± 0.00
*P. harmala*	Nitrariaceae	Fruit	966,510,482	0.46 ± 0.12	39.30 ± 0.20	155.29 ± 0.20
*J. conglomeratus*	Juncaceae	Fruit	966,510,126	7.19 ± 0.89	45.66 ± 0.10	46.54 ± 0.10

### Plant extracts preparation

The collection of plant materials complied with institutional guidelines, and whole plant materials were wild type requiring no licenses for the application. The fresh selected parts of each plant were washed by the distilled water, shade-dried and then powdered in a mechanical mill. Afterward, 10 g of powdered materials was soaked into 170 mL methanol and chloroform, separately. The plugged flasks of samples solution were placed at room temperature for 48 h by persistent shaking. The crude solutions were filtered through glass funnel and then dried via a rotary vacuum evaporator at 40 °C temperature. Finally, the extracts were filter sterilized by a 0.22 µm Ministart (Sartorius) and stored at 4 °C before utilization [16].

### Essential oils separation

The powdered samples (75 g) were exposed to hydro-distillation for 4 h, using a Clevenger-type apparatus. The essential oils were dehydrated by sodium sulfate anhydrous and stored at 4 °C before GC/MS analysis [17–19].

### Gas chromatography coupled to mass spectrometry (GC/MS) analysis

GC/MS analysis was performed on an Agilent Technologies 7890A (GC) coupled with Agilent Technologies 5975C, equipped with a fused silica capillary HP-5MS column (30 m × 0.25 mm iD, film thickness 0.25 µm). The oven temperature was increased from 50 to 220 °C at a speed of 15 °C min^−1^, retained at 220 °C for 7 min; and then incremented to 260 °C at a speed of 15 °C min^−1^. Transfer line temperature was 250 °C. Helium was used as the carrier gas, at a flow speed of 1 mL min^−1^. The inlet temperature was 280 °C.

### Antioxidant assays

Dry samples (0.5 g) were homogenized in the extraction buffer (1 mL) containing; EDTA (1 mM), PVP (1%) and sodium phosphate buffer (50 mM, pH = 7) by mortar and pestle. Afterwards, the homogenates were centrifuged (Eppendorf centrifuge 5430R) at 10,000 g for 15 min. Finally, the supernatant fractions were utilized for the measurement of protein content and enzyme activities [20].

### Measurement of catalase (CAT)

Catalase was examined via evaluating the primary rate of disappearance of H_2_O_2_, according to the Chance and Meahly [21] method. The reaction mixture, including phosphate buffer (2.5 mL, 50 mM, pH = 7), H_2_O_2_ (0.1 mL, 1%) and enzyme extracts (50 µL), was diluted in order to keep the measurements within the linear range of the analysis. The absorbance of the reaction mixtures was recorded at 240 nm via spectrophotometer (Biochrom WPA Biowave II UV/Visible), in which the reduction in the absorbance at 240 nm was because of the reduction of H_2_O_2_. The activity was stated as µmole activity mg^−1^ protein.

### Measurement of guaiacol peroxidase

Guaiacol peroxidase (GPX) activity was studied according to the Upadhyaya et al. [22] method. The reaction combination included phosphate buffer (2.5 mL, 50 mM, pH = 7), H_2_O_2_ (1 mL, 1%), guaiacol (1 mL, 1%), and enzyme extracts (20 µL). The absorbance of the reaction mixtures was recorded at 470 nm via spectrophotometer (Biochrom WPA Biowave II UV/Visible), and the increment in absorbance at 470 nm was followed for 1 min. The activity was stated as mmole activity mg^−1^ protein.

### Measurement of protein

Protein concentrations were specified based on the Bradford [23] method, by Bovine Serum Albumin (BSA), as standard protein.

### 2, 2- Di-Phenyl-1-Picryl Hydrazyl (DPPH) scavenging

The antiradical activity of the methanol extract of samples was evaluated using a spectrophotometer, via Liyana-Pathirana and Shahidi [24] method. A solution of 0.135 mM DPPH in methanol was made, and then, 1.0 mL of this solution was blended with 1.0 mL of the methanol extract of the samples in methanol including 40–270 µg of the methanol extract. The reaction mixtures were vortexed completely and placed for 30 min in the dark at room temperature. The mixtures absorbance was recorded spectrophotometrically at 517 nm. Ascorbic acid was utilized as a reference. The capability to scavenge DPPH radical was computed using the following equation:

$${\text{DPPH}}\;{\text{scavenging}}\;{\text{assay}}\; ( {\text{\% )}}\;{ = }\; [ ( {\text{Abs}}_{\text{control}} \; - \;{\text{Abs}}_{\text{sample}} )/{\text{Abs}}_{\text{control}} ]\; \times \;100.$$

where, Abs_control_ is the absorbance of DPPH radical + methanol; and Abs_sample_ is the absorbance of DPPH radical + samples methanol extract. The radical scavenger activity was stated as the extent of antioxidants required to reduce the primary DPPH absorbance by 50% (IC_50_). The IC_50_ amount for any sample was calculated graphically through plotting the percentage of disappearance of DPPH as a function of the sample concentration.

### Phytochemical analysis

Total Phenolic Content (TPC) of the test samples was assayed using Yu et al. [25] Folin–Ciocalteu method, utilizing gallic acid as the standard. Briefly, double distilled water (900 µL) was added to the methanolic solution of test samples (100 µL, 100 µg mL^−1^). Then, Folin–Ciocalteu reagent (500 µL) was added, followed by the addition of sodium carbonate (1.5 mL, 20%). The volume of the mixture was reached to 10 mL by the distilled water. The mixture was afterward incubated at room temperature for 2 h. After that, the absorbance was assayed via spectrophotometer (Biochrom WPA Biowave II UV/Visible) at 725 nm. The same method was used for the standard solutions of gallic acid. Based on the evaluated absorbance, the concentration of phenolic content was determined from the calibration line. Finally, the total phenolic content of methanol extracts was stated as mg Gallic Acid Equivalents (GAE) g^−1^ dry matter.

In order to determine the flavonoid content, the colorimetric aluminum chloride method was utilized [26]. Each sample in methanol (0.5 mL, 1:10 g mL^−1^) was blended with methanol (1.5 mL), potassium acetate (0.1 mL, 1 M), aluminum chloride (0.1 mL, 10%), and the distilled water (2.8 mL). Then, the extracts were placed at room temperature for 30 min. Afterwards, the absorbance of the reactions was recorded using spectrophotometer (Biochrom WPA Biowave II UV/Visible) at 415 nm. The calibration curve was plotted through making quercetin solutions (12.5 to 100 µg mL^−1^) in methanol. Finally, the total flavonoid content was stated as mg of quercetin equivalents g^−1^ of dry sample.

### Antibacterial screening

Microorganisms *Staphylococcus aureus* PTCC 1431, *Bacillus subtilis* PTCC 1023, *Pseudomonas aeruginosa* PTCC 1074, *Escherichia coli* PTCC 1330, *Pseudomonas syringae* subsp*. Syringae* ICMP 5089, *Pseudomonas viridiflava* ICMP 2848, *Rathayibacter toxicus* ICMP 9525, and *Xanthomonas campestris* pv. *Campestris* ICMP 13 were obtained from the Sari Agricultural and Natural Resources University (SANRU) microbiology laboratory.

The antibacterial effect of the methanol and chloroform extracts of the samples was assessed with the disk diffusion method utilizing Mueller–Hinton agar [17, 33], and investigation of inhibition zones of the extracts. The filter paper discs of 6 mm diameter (Padtan, Iran) were sterilized then impregnated with 25 µL of methanol and chloroform extracts, separately. The sterile impregnated discs were put on the agar surface by the flamed forceps and softly compressed down to ensure perfect contact of the discs with the agar surface. The incubation condition was 37 °C for quality control strains and 27 °C for plant bacteria for 24 h. All trials were performed in triplicate and the results were stated as mean ± SD.

The antibacterial activity was evaluated by determining the Minimum Inhibitory Concentration (MIC), employing broth dilution method [18]. Each strain was tested with an extract serially diluted in Luria broth, to obtain concentrations ranging from 100 to 0.8 µg mL^−1^. The samples were thereafter stirred, inoculated with 50 µg mL^−1^ of physiologic solution containing 5 × 10^8^ microbial cells, and incubated at 37 °C for quality control strains and 27 °C for plant bacteria for 24 h. A number of wells were reserved on each plate for sterility control (no inoculum), inoculum viability (no extract added), and the positive control (Gentamicin). The MIC was stated as the lowest concentration of extract that visibly inhibited the growth of bacterial spots. The assays were performed in triplicate.

To determine the Minimum bactericidal Concentration (MBC), 10 µL of aliquot broth were taken from each well, and plated in Mueller–Hinton agar for 24 h at 37 °C for quality control strains, and 27 °C for plant bacteria. The MBC represents the concentration required to kill 99.9% or more of the initial inoculum [18]. The assays were performed in triplicate.

### Antifungal effect

The following microorganisms were utilized: *Fusarium oxysporum*, *Pyricularia oryzae*, and *Botrytis cinerea*.

The antifungal property of the methanol and chloroform extracts was examined with the agar-well diffusion method [16]. Potato Dextrose Agar (PDA) was seeded by tested fungus. Sterile paper discs of 6 mm diameter (Padtan, Iran) were impregnated by 25 µL of the methanol and chloroform extracts of samples, separately. The sterile impregnated discs were put on the level of the seeded agar plate. The incubation conditions utilized were 28 °C and 70% RH for 12–14 days for *Pyricularia oryzae* and 7–9 days for *Botrytis cinerea*, and *Fusarium oxysporum*. The antifungal activity was visualized as a zone of inhibition of fungal growth around the paper disc and the results were stated as mean ± SD after three repetitions. Pathogen grown on PDA without plant extract was utilized as control.

### Statistical analysis

Methanol and chloroform extracts tested in triplicate for chemical analysis and bioassays. The obtained data were exposed to Analysis of Variance (ANOVA), following a completely randomized design to determine the Least Significant Difference (LSD) at P < 0.05 by SPSS statistical software package (SPSS v. 11.5, IBM Corporation, Armonk, NY, USA). All results were stated as mean ± SD. Independent-sample t-test was used for selected comparisons between samples. Alpha value was set a priori at P < 0.05.

## Results and discussion

### Essential oils compounds

As *S. flavescens* and *P. harmala* plants showed the best antimicrobial activities, they were selected for GC/MS analysis to identify the effective compounds. The results are shown below, separately.

### *S. flavescens*

Thirty-three constituents were recognized in the essential oil of *S. flavescens* aerial parts, representing 93.70% of the total essential oil. The essential oil combinations are listed in the order of their elution on the HP-5MS column as follows: Decane (0.44%), p-Cymene (0.31%), γ-Terpinene (0.39%), α-Terpinolene (0.26%), Terpinen-4-ol (0.35%), 4-isopropyl-2-cyclohexenone (0.46%), 1,6- cyclodecadiene (4.59%), Benzaldehyde, 4-(1-methylethyl)- (1.12%), Thymol (1.70%), Carvacrol (0.26%), β-Damascenone (0.91%), Caryophyllene (1.09%), Nerylacetone (0.44%), 2,6,10,14-Tetramethylheptadecane (0.49%), Alloaromadendrene (6.59%), α-curcumene (0.55%), β-Ionone (0.55%), 3,5-Di-tert-butylphenol (0.48%), Germacrene D (0.35%), Dodecanoic acid (3.37%) (+)-spathulenol (15.39%), Caryophyllene oxide (1.43%), Ledene (0.67%), Tetradecanoic acid (1.13%), 6,10,14-trimethylpentadecan-2-one (5.15%), Diisobutyl phthalate (0.65%), methyl 14-methylpentadecanoate (1.99%), *n*-Hexadecanoic acid (8.86%), Butyl 2-ethyl hexyl phthalate (1.20%), Squalene (8.87%), Ethyl linoleolate (4.99%), Neophytadiene (17.61%), and Linoleic acid (1.06%).

GC/MS analysis showed that the main components of the essential oil were Neophytadiene (17.61%), Spathulenol (15.39%), and Squalene (8.87%).

### *P. harmala*

Eighteen components were identified in the essential oil of *P. harmala* fruits representing 91.76% of the total essential oil. The essential oil compounds are listed in the order of their elution on the HP-5MS column as follows: Decane (1.05%), m-Cymene (0.78%), γ-Terpinene (0.74%), 4-carvomenthenol (1.52%), 4-isopropyl-2-cyclohexenone (0.81%), Cuminaldehyde (2.58%), Thymol (2.46%), β-caryophyllene (1.44%), 6,10-dimethyl-5,9-undecadiene-2-one (0.88%), Alloaromadendrene (5.00%) (-)-Spathulenol (37.83%) (+)-Aromadendrene (1.07%), β-oplopenone (0.39%), Methyl palmitate (1.14%), *n*-Hexadecanoic acid (13.21%), Methyl linoleate (1.04%), Linoleic acid (11.08%), and Elaidic acid (8.72%).

GC/MS analysis showed that the main components of the essential oil were Spathulenol (37.83%), *n*-Hexadecanoic acid (13.21%), and Linoleic acid (11.08%).

### Protein content and enzymes activity

**Fig. 1:**
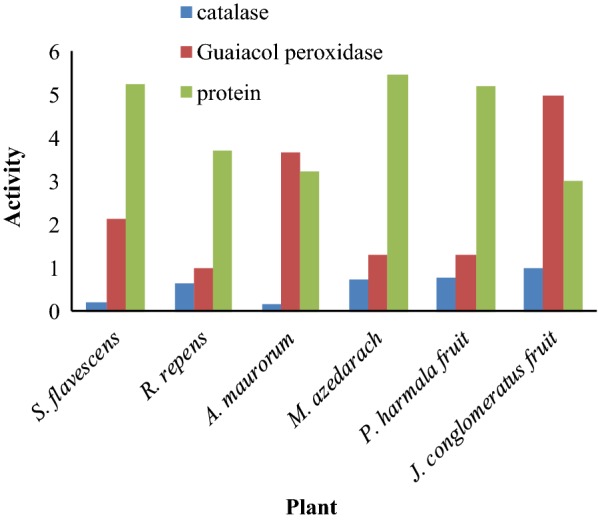
Enzymes activity and protein content

Plants have evolved antioxidant pathways that are usually sufficient to protect them from oxidative injury during periods of natural growth and moderate stress. Both enzymatic and non-enzymatic systems protected tissue from the activated oxygen species, produced as the result of external environmental stresses, such as dryness, chilling and air pollution. Certain enzymatic antioxidant defense systems contain Super Oxide Dismutase (SOD), Catalase (CAT), and Guaiacol Peroxidase (GPX) [27]. In this research, the activity of 2 enzymes (CAT and GPX) was evaluated. Moreover, protein content was measured by bovine serum albumin as a standard. The results are exhibited in Fig. 1. As shown, the maximum and the minimum activities of catalase were found in *J. conglomeratus* and *S. flavescens* plants, respectively. Besides, guaiacol peroxidase activity assay indicated that *J. conglomeratus* plant had the highest activity. Furthermore, the minimum guaiacol peroxidase activity was related to *R. repens* plant. Moreover, the maximum and the minimum protein contents were observed in *M. azedarach* fruit and *J. conglomeratus* plant, respectively.

### DPPH radical scavenging effect

The effect of antioxidants on DPPH. was assumed to be because of their hydrogen donating capability [28]. Table 1 shows the DPPH radical scavenging effect of the tested plants. As presented, the highest free radical scavenging capacity of the plants was determined in *P. harmala* extract with an IC_50_ value of 0.46 ± 0.12 µg mL^−1^.

### Total phenol and flavonoid content of the extracts

Plants have unlimited capability to produce aromatic secondary metabolites, which most of them are phenols or their oxygen-substituted derivatives. Key subclasses in this set of compounds contain phenols, phenolic acids, quinones, flavones, flavonoids, flavonols, tannins, and coumarins. These collections of compounds indicate antimicrobial activity and apply as plant defense mechanisms versus pathogenic microorganisms. Phenolic toxicity to microorganisms is because of the number of hydroxyl groups and site(s) existing in the phenolic compounds. Phenolic compounds cause cell membrane disruption, increase of ion permeability and leakage of vital intracellular constituents or impairment of bacterial enzyme systems in pathogenic microorganisms [34, 35].

It has been recognized that the antioxidant effect of the flavonoids and their effectiveness on human health and nutrition are considerable. Chelating or scavenging procedures are the action mechanism of flavonoids [29]. The evaluation of total flavonoid content was based on the determining the absorbance amount of tested plant solutions reacting with aluminum chloride reagent, and comparing with the standard solution of quercetin equivalents. The standard curve of quercetin was performed utilizing quercetin concentration ranging from 12.5 to 100 µg mL^−1^. The following equation stated the absorbance of the standard solution of quercetin as a function of concentration:

$${\text{Y}}=0.0056{\text{x}}\;{ + }\;0.1764,\;{\text{R}}^{2} = 0.9878$$

where, x is the absorbance and Y is the quercetin equivalent (mg g^−1^). The flavonoid content of samples is shown in Table 1. As shown, the highest phenol content was determined in *A. maurorum*, *P. harmala* and *S. flavescens* extracts with a value of 45.43, 39.3 and 39.07 mg of quercetin equivalents g^−1^ of dry matter, respectively.

Phenolic compounds gained from plants are a class of secondary metabolites, acting as an antioxidant or free radical terminators. Therefore, it is necessary to evaluate the total content of phenols in the tested plants [30]. The designation of the total phenolic amount was based on the absorbance amount of sample solutions (100 µg mL^−1^) reacting with Folin-Ciocalteu reagent, and comparing with the standard solution of gallic acid equivalents. The standard curve of gallic acid was performed utilizing gallic acid concentration ranging from 12.5 to 100 µg mL^−1^. The following equation stated the absorbance of the gallic acid standard solution as a function of concentration:

$${\text{Y}}\;{ = }\; 0. 0 9 5 4 {\text{x}}\;{ + }\; 0. 1 9 6 , {\text{ R}}^{2}\;{ = }\; 0.9973$$

where, x is the absorbance and Y is the gallic acid equivalent (mg g^−1^). The phenol content of the samples is presented in Table 1. As shown, the highest phenol content was determined in *P. harmala* and *A. maurorum* extracts with a value of 155.29 ± 0.20 and 146.71 ± 0.02 mg Gallic Acid Equivalents (GAE) g^−1^ dry matters, respectively.

### Antibacterial screening

**Fig. 2:**
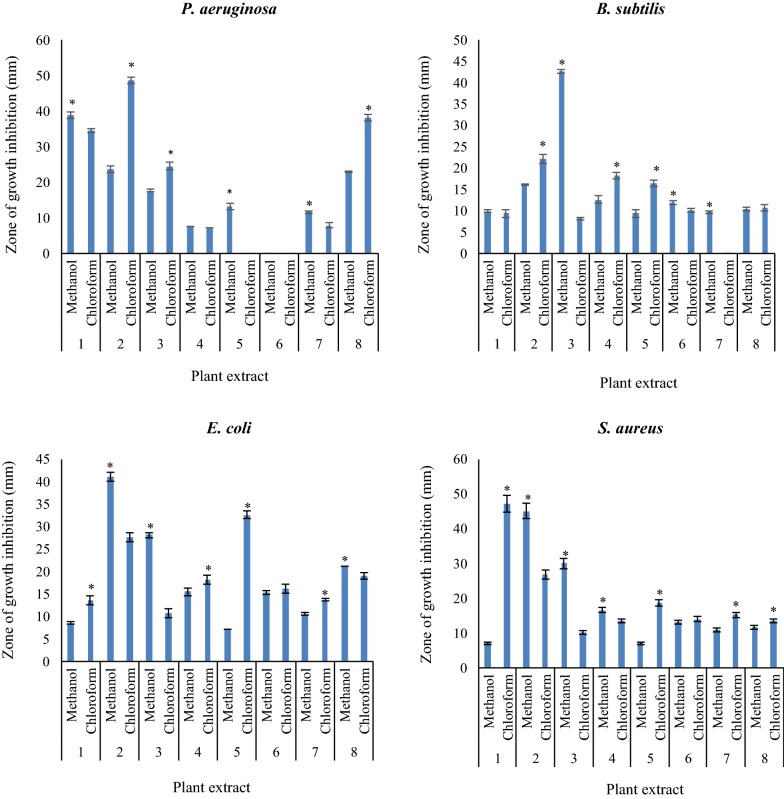
The antibacterial activity of methanolic and chloroformic extracts including 1: *S. flavescens*; 2: *P. harmala* fruit; 3: *P. harmala*; 4: *R. repens*; 5: *M. azedarach*; 6. *J. conglomeratus* fruit; 7: *A. maurorum*; 8: *J. conglomeratus* on standard bacteria strains. Data were exposed to Analysis of Variance (ANOVA), following a completely randomized design to determine the Least Significant Difference (LSD) at P < 0.05 by SPSS statistical software package (SPSS v. 11.5, IBM Corporation, Armonk, NY, USA). All consequences were stated as mean ± SD. Also, * using independent t-test between the two groups

**Fig. 3:**
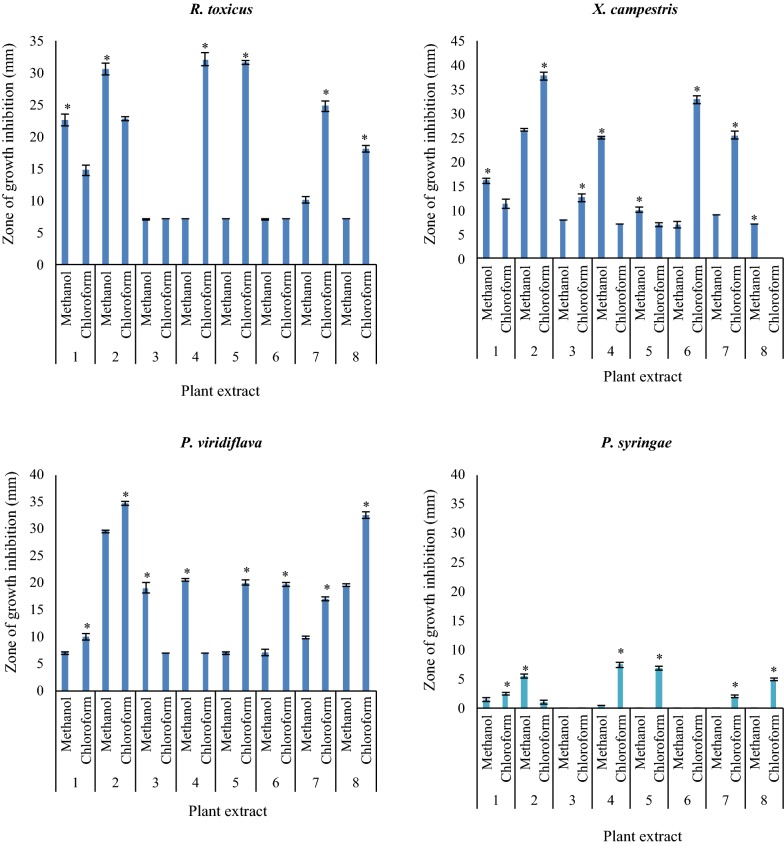
The antibacterial activity of methanolic and chloroformic extracts including 1: *S. flavescens*; 2: *P. harmala* fruit; 3: *P. harmala*; 4: *R. repens*; 5: *M. azedarach*; 6. *J. conglomeratus* fruit; 7: *A. maurorum*; 8: *J. conglomeratus* on plant bacteria strains. Data were exposed to Analysis of Variance (ANOVA), following a completely randomized design to determine the Least Significant Difference (LSD) at P < 0.05 by SPSS statistical software package (SPSS v. 11.5, IBM Corporation, Armonk, NY, USA). All consequences were stated as mean ± SD. Also, * using independent t-test between the two groups

The antibacterial activity of methanolic and chloroformic extracts including *A. maurorum*, *S. flavescens*, *R. repens*, *M. azedarach*, *P. harmala* and *J. conglomeratus* in different concentrations (0.01, 0.03, 0.06, 0.12, 0.25 and 0.5 ppm) were tested versus 3 g-positive (*B. subtilis*, *S. aureus*, *R. toxicus*) and 5 g-negative (*P. aeruginosa*, *E. coli*, *X. campestris*, *P. viridiflava*, *P. syringae*) bacteria. The results at 0.5 ppm are shown in Figs. 2, 3. In addition, as in other concentrations, similar results were observed, for simplifying the discussion we considered only 0.5 ppm concentration. As shown in Fig. 2, methanolic extracts of *S. flavescens*, *P. harmala* fruit and *J. conglomeratus* and chloroformic extracts of *P. harmala* fruit, *S. flavescens*, and *P. harmala* showed the maximum antibacterial activity on *P. aeruginosa,* respectively. Furthermore, methanolic extract of *J. conglomeratus* fruits and chloroformic extracts of *M. azedarach* and *J. conglomeratus* fruit had no antibacterial effect on *P. aeruginosa* (Fig. 2a). The methanolic extract of *P. harmala* and chloroformic extracts of *P. harmala* fruit, *R. repens*, and *M. azedarach* had the maximum antibacterial activity against *B. subtilis*, respectively. Besides, chloroformic extract of *A. maurorum* extract had no antibacterial activity on *B. subtilis* (Fig. 2b). The methanolic extracts of *P. harmala* fruit, *P. harmala*, and *J. conglomeratus* and chloroformic extracts of *M. azedarach* and *P. harmala* fruit indicated the maximum antibacterial activity on *E. coli*, respectively (Fig. 2c). Moreover, the methanolic extracts of *P. harmala* fruit, the aerial part and chloroformic extracts of *S. flavescens* and *P. harmala* fruit had the maximum antibacterial activity on *S. aureus*, respectively (Fig. 2d). Moreover, the antibacterial activity of tested plants on plant bacteria strains is shown in Fig. 3. As indicated, methanolic extracts of *P. harmala* fruit and *S. flavescens* and chloroformic extracts of *R. repens* and *M. azedarach* showed the maximum antibacterial activity against *R. toxicus*, respectively (Fig. 3a). Furthermore, methanolic extracts of *R. repens* and *P. harmala* fruit and chloroformic extracts of *P. harmala* fruit, *J. conglomeratus* fruit and, *A. maurorum* presented the maximum antibacterial activity against *X. campestris*, respectively (Fig. 3b). The methanolic extract of *P. harmala* fruit and chloroformic extracts of *P. harmala* and *J. conglomeratus* displayed the maximum antibacterial activity on *P. viridiflava* (Fig. 3c). Besides, the methanolic extracts of *S. flavescens*, *P. harmala* fruit and *R. repens* and chloroformic extracts of *R. repens* represented the maximum antibacterial activity on *P. syringae*, respectively. However, the methanolic extract of *J. conglomeratus* fruit showed no antibacterial activity (Fig. 3d).

In order to compare the antibacterial activities of methanolic and chloroform extracts, independent-sample t-test was used, indicated with asterisk in Figs. 2, 3. For example, in Fig. 2a, methanolic and chloroform extracts of plants 1, 2, 3, 5, 7 and 8 showed significant differences on *Pseudomonas bacteria*. In Fig. 2b, methanolic and chloroform extracts of plants 2, 3, 4, 5, 6 and 7 displayed significant differences on *B. subtilis*. In Fig. 2c, methanolic and chloroform extracts of plants 1, 2, 3, 4, 5, 7 and 8 exhibited significant differences on *E. coli*. In Fig. 2d, methanolic and chloroform extracts of plants 1, 2, 3, 4, 5, 7 and 8 exhibited significant differences on *S. aureus*. While in Fig. 3a, methanolic and chloroform extracts of plants 1, 2, 4, 5, 7 and 8 presented significant differences on *R. toxicu*, in Fig. 3b, methanolic and chloroform extracts of plants 1, 2, 3, 4, 5, 6, 7 and 8 presented significant differences on *X. campestris*. Besides, in Fig. 3c, methanolic and chloroform extracts of plants 1, 2, 3, 4, 5, 6, 7 and 8 showed significant differences on *P. viridiflava*, whereas in Fig. 3d, methanolic and chloroform extracts of plants 1, 2, 3, 4, 5, 6, 7 and 8 showed significant differences on *P. syringae*.

Furthermore, Tables 2, 3 illustrate the MIC and MBC values of the methanolic and chloroformic extracts of the tested medicinal plants against bacteria, respectively. The methanolic extract of *P. harmala* fruits showed the maximum activity against *S. aureus* and *E. coli* with MIC = 1.56 µg mL^−1^. In addition, chloroformic extracts of *S. flavescens* and *P. harmala* fruit indicated maximum activity against *S. aureus* and *P. aeruginosa* with MIC = 1.56 µg mL^−1^, respectively.

**Table 2:** The Minimal Inhibitory Concentration (MIC, µg mL^−1^) and the Minimum Microbicidal Concentration (MBC, µg mL^−1^) of the methanolic extract of the tested medicinal plants against bacteria

Strain	MIC (MBC)						Gentamicin
	*S. flavescens*	*R. repens*	*A. maurorum*	*M. azedarach*	*P. harmala* fruit	*J. conglomeratus* fruit	
*B. subtilis*	–^a^	50 (100)	–	–	50 (100)	100 (−)	6.24
*S. aureus*	–	50 (100)	100 (−)	–	1.56 (3.12)	50 (100)	3.12
*R. toxicus*	25 (50)	–	–	–	12.5 (25)	–	NS^b^
*E. coli*	–	50 (100)	–	–	1.56 (3.12)	50 (100)	1.56
*P. aeruginosa*	12.5 (25)	–	100 (−)	50 (100)	25 (50)	–	12.48
*P. syringae*	–	100 (−)	–	–	100 (−)	–	NS
*P. viridiflava*	–	25 (50)	–	–	25 (50)	–	NS
*X. campestris*	50 (100)	25 (50)	–	–	25 (50)	–	NS

^a^No inhibition with the highest concentration in the test conditions

^b^Not specified

**Table 3:** The Minimal Inhibitory Concentration (MIC, µg mL^−1^) and the Minimum Microbicidal Concentration (MBC, µg mL^−1^) of the chloroformic extract of the tested medicinal plants against bacteria

Strain	MIC (MBC)						Gentamicin
	*S. flavescens*	*R. repens*	*A. maurorum*	*M. azedarach*	*P. harmala* fruit	*J. conglomeratus* fruit	
*B. subtilis*	–^a^	50 (100)	–	50 (100)	25 (50)	–	6.24
*S. aureus*	1.56 (3.12)	50 (100)	50 (100)	50 (100)	25 (50)	50 (100)	3.12
*R. toxicus*	50 (100)	12.5 (25)	25 (50)	12.5 (25)	25 (50)	25 (50)	NS^b^
*E. coli*	100 (100)	50 (100)	50 (100)	12.5 (25)	25 (50)	50 (100)	1.56
*P. aeruginosa*	12.5 (25)	–	–	–	1.56 (3.12)	–	12.48
*P. syringae*	–	100 (−)	–	100	–	–	NS
*P. viridiflava*	–	–	50 (100)	25	12.5 (25)	50 (100)	NS
*X. campestris*	100 (100)	–	25 (50)	–	12.5 (25)	12.5 (25)	NS

^a^No inhibition with the highest concentration in the test conditions

^b^Not specified

### Antifungal activity

The antifungal properties of the methanolic and chloroformic extracts were tested using the agar well diffusion method. The results of the experiments showed that none of the tested plants had antifungal activity.

The use of herbal extracts as antioxidant and antimicrobial agents has two separate advantages: the natural origin and the related low risk. This means that they cause fewer side effects for people and the environment [31]. Based on the results, methanolic and chloroformic extracts of *P. harmala* fruit showed the maximum antibacterial activity against most of the tested bacteria pathogens, attributable to higher content of phenolic and flavonoid compounds. In addition, our findings were in agreement with those of Hayet et al. [9] and Guergour et al. [32]. Methanolic and chloroformic extracts of *S. flavescens* indicated the maximum antibacterial activity against *P. aeruginosa* and *S. aureus*, respectively. Our findings were in according with Han and Guo [10] and Yang et al. [31]. Chloroformic extract of *M. azedarach* represented the maximum antibacterial activity on *E. coli*, in accordance with Sen and Batra [11]. methanolic and chloroformic extracts of *A. maurorum* indicated antibacterial activity against all tested bacteria pathogens, in agreement with the study of Ahmad et al. [12].

## Conclusion

In this work, the antimicrobial and antioxidant activities of extracts of some plants used in Iranian folklore medicine were reported. Based on the results, methanolic and chloroformic extracts of *P. harmala* fruit showed the maximum antibacterial activity against most of the tested bacteria pathogens, attributable to higher content of phenolic and flavonoid compounds. According to the obtained results, a high resolution GC/MS method reported for the evaluation of the constituents of *P. harmala* and *S. flavescens* plants, while in both plants, Spathulenol was the main component of the essential oil. Furthermore, in this study, the antibacterial and antifungal activities of medicinal plants extracts on plant bacteria and fungi strains were evaluated for the first time. Furthermore, antioxidant assays including measurement of catalase, guaiacol peroxidase and protein were reported for the first time in this study.

In conclusion, the results confirmed the traditional use of the herb against antimicrobial diseases. These plants could act as a potential antimicrobial agent; however, further studies are required for them to be safely used in the control of disease and pests.

## Data Availability

All data and materials are all provided.
